# Somatic Mutations in DNA Mismatch Repair Genes, Mutation Rate and Neoantigen Load in Acute Lymphoblastic Leukemia

**DOI:** 10.3390/ph18091405

**Published:** 2025-09-18

**Authors:** Diana Karen Mendiola-Soto, Laura Gómez-Romero, Juan Carlos Núñez-Enríquez, Janet Flores-Lujano, Elva Jiménez-Hernández, Aurora Medina-Sansón, Vilma Carolina Bekker-Méndez, Minerva Mata-Rocha, María Luisa Pérez-Saldívar, David Aldebarán Duarte-Rodríguez, José Refugio Torres-Nava, José Gabriel Peñaloza-González, Luz Victoria Flores-Villegas, Raquel Amador-Sánchez, Martha Margarita Velázquez-Aviña, Jorge Alfonso Martín-Trejo, Laura Elizabeth Merino-Pasaye, Karina Anastacia Solís-Labastida, Rosa Martha Espinosa-Elizondo, Carlos Jhovani Pérez-Amado, Didier Ismael May-Hau, Omar Alejandro Sepúlveda-Robles, Haydee Rosas-Vargas, Juan Manuel Mejía-Aranguré, Silvia Jiménez-Morales

**Affiliations:** 1 Laboratorio de Innovación y Medicina de Precisión, Núcleo A, Instituto Nacional de Medicina Genómica, Ciudad de Mexico 14610, Mexico; krn_mdl@hotmail.com (D.K.M.-S.); didier_may@outlook.com (D.I.M.-H.); 2 Programa de Doctorado en Ciencias Biomédicas, Universidad Nacional Autónoma de México, Ciudad de Mexico 04510, Mexico; 3 Departamento de Bioinformática, Instituto Nacional de Medicina Genómica, Ciudad de Mexico 14610, Mexico; lgomez@inmegen.gob.mx; 4 Tecnológico de Monterrey, School of Medicine and Health Sciences, Ciudad de Mexico 14380, Mexico; 5 Unidad de Investigación Médica en Epidemiología Clínica, Unidad Médica de Alta Especialidad (UMAE), Hospital de Pediatría “Dr. Silvestre Frenk Freund”, Centro Médico Nacional (CMN) “Siglo XXI”, Instituto Mexicano del Seguro Social (IMSS), Ciudad de Mexico 06720, Mexico; 6 Unidad de Investigación Médica en Epidemiología Clínica, Unidad Médica de Alta Especialidad (UMAE), Hospital de Pediatría, Centro Médico Nacional (CMN) “Siglo XXI”, Instituto Mexicano del Seguro Social (IMSS), Ciudad de Mexico 06720, Mexico; 7 Servicio de Oncología, Hospital Pediátrico “Moctezuma”, Secretaría de Salud de la Ciudad de Mexico (SEDESA), Ciudad de Mexico 15530, Mexico; 8 Departamento de Hemato-Oncología, Hospital Infantil de Mexico “Federico Gómez”, Secretaría de Salud (SS), Ciudad de Mexico 06720, Mexico; 9 Unidad de Investigación Médica en Inmunología e Infectología, Hospital de Infectología “Dr Daniel Méndez Hernández”, Centro Médico Nacional (CMN) “La Raza”, Instituto Mexicano del Seguro Social (IMSS), Ciudad de Mexico 02990, Mexico; bekkermendez@yahoo.com; 10 Laboratorio de Biología Molecular, Unidad Médica de Alta Especialidad (UMAE), Hospital de Pediatría, Centro Médico Nacional (CMN) “Siglo XXI”, Instituto Mexicano del Seguro Social (IMSS), Ciudad de Mexico 06720, Mexico; 11 División de Desarrollo de la Investigación en Salud, Coordinación de Investigación en Salud, Centro Médico Nacional (CMN) “Siglo XXI”, Instituto Mexicano del Seguro Social (IMSS), Ciudad de Mexico 06720, Mexico; 12 Servicio de Onco-Pediatría, Hospital Juárez de México, Secretaría de Salud (SSA), Ciudad de Mexico 07760, Mexico; 13 Servicio de Hematología Pediátrica, Centro Médico Nacional “20 de Noviembre”, Instituto de Seguridad y Servicios Sociales de los Trabajadores del Estado, Ciudad de México 03104, Mexico; 14 Hospital General Regional 1 “Dr. Carlos McGregor Sánchez Navarro”, IMSS, Ciudad de México 03103, Mexico; 15 Servicio de Hematología, Unidad Médica de Alta Especialidad (UMAE), Hospital de Pediatria, Centro Médico Nacional Siglo XXI, IMSS, Ciudad de México 06720, Mexico; 16 Servicio de Hematología Pediátrica, Centro Médico Nacional (CMN) “20 de Noviembre”, Instituto de Seguridad Social al Servicio de los Trabajadores del Estado (Instituto de Seguridad Social al Servicio de los Trabajadores del Estado (ISSSTE)), Ciudad de México 03104, Mexico; 17 Servicio de Hematología Pediátrica, Hospital General de México, Secretaría de Salud (SSA), Ciudad de México 06720, Mexico; 18 Unidad de Investigación Médica en Genética Humana, Unidad Médica de Alta Especialidad (UMAE), Hospital de Pediatría, Centro Médico Nacional (CMN) “Siglo XXI”, Instituto Mexicano del Seguro Social (IMSS), Ciudad de Mexico 06720, Mexicohayrov@gmail.com (H.R.-V.); 19 Laboratorio de Genómica Funcional del Cáncer, Instituto Nacional de Medicina Genómica (INMEGEN), Ciudad de Mexico 14610, Mexico; 20 Facultad de Medicina, Universidad Nacional Autónoma de Mexico (UNAM), Ciudad de Mexico 04360, Mexico

**Keywords:** acute lymphoblastic leukemia, neoantigens, exome sequencing, HLA-A*02:01, HLA-B*39:05, HLA-C*07:01

## Abstract

**Background/Objectives**: During cancer development, tumor cells accumulate somatic mutations, which could generate tumor-specific neoantigens. The aberrant protein can be recognized by the immune system as no-self, triggering an immune response against cells expressing this aberrant protein which could mediate tumor control or rejection. Since the expression of this mutated protein is exclusive to tumor cells, great efforts are being made to identify neoantigens of relevance in the development of new cancer treatment strategies. In comparison to adulthood tumors, pediatric malignancies present fewer mutations and thus fewer potential neoantigens. Acute lymphoblastic leukemia (ALL) is the most common pediatric malignancy worldwide that can be benefited by the identification of neoantigens for immunotherapy approaches, the landscape of neoantigens in ALL is not well known, therefore the aim of our study was to identify potential neoantigens in ALL pediatric patients. **Methods**: To identify neoantigens in ALL, whole-exome sequencing of matched tumor-normal cells from pediatric cases was performed, with these data HLA-I alleles predicted and somatic mutations identified to propose potential neoantigens based on binding affinity of mutated peptide-HLA-I. **Results**: We found a strong correlation between tumor mutational burden (TMB) and neoantigen load (*p* < 0.001) but no correlation with prognosis. Furthermore, TMB and neoantigens were greater in ALL patients with at least one mutated DNA mismatch repair gene (*p* < 0.001). Also, differences between B- and T-cell ALL were found but statistical significance did not remain after permutation. **Conclusions**: The presence of neoantigens in pediatric cases with ALL makes the neoantigen-based immunotherapy a promising new strategy for the treatment of this malignancy, especially for patients with relapse.

## 1. Introduction

Acute lymphoblastic leukemia (ALL) is the most common pediatric malignancy worldwide, accounting for 30% of all pediatric malignancies in developed countries and up to 50% in developing ones [1,2]. It is also well documented that ALL displays a differential incidence rate among ethnic groups, being higher in Hispanics (~45 cases per million) than in Caucasians and Asians. ALL in the Mexican population not only shows one of the highest incidence rates (53 cases per million), but also has low frequency of good prognosis biomarkers [3,4]. For instance, around 10% of pediatric ALL cases from Mexico are positive to ETV6-RUNX1 but this biomarker accounts for ~25% of cases in other populations [5,6,7,8]. Furthermore, approximately 40% of Mexican pediatric cases die after an average follow-up of 3.9 years and more than a half of these deaths are related to relapse [9,10]. A plethora of evidence has highlighted the importance of acquired somatic mutations during cancer development, which is also considered a potential mechanism associated with relapse and overall survival (OS) rate in ALL [11]. Furthermore, acquired genetic alterations could result in the production of tumor-specific antigens or neoantigens, which might trigger an immune response against tumor cells expressing those mutated peptides and control tumor progression [12,13]. As the mismatch repair (MMR) system is responsible for recognizing and repairing incorrectly paired nucleotides that arise in DNA, its deficiency or inactivation may drive the rise in mutational burden. Mutations in DNA MMR genes have been previously related with risk and recurrence in ALL, which also impact neoantigen renewal [14,15,16,17]. Triggering the immune system through the use of neoantigens is constantly being highlighted as an important therapeutic target for cancer treatment [18,19,20,21]. As ALL emerges in lymphoid organs, where anti-tumor immune responses are typically initiated, its origin can cause immune sensing mechanisms failure or suppression of immune response, which might suggest that this hematological malignancy is poorly immunogenic. Nevertheless, the immune responsiveness in allogenic hematopoietic stem cell transplantation and in immunotherapy for relapsed or refractory ALL set the basis to hypothesize that leukemic cells could express new immunogenic specific peptides [22]. Even though it has been described that most hematologic malignancies have fewer protein coding mutations which generate fewer potential neoantigens than solid tumors, targeting a single high-quality neoantigen can be enough for disease control or even cure [18,23,24]. Supporting this hypothesis, a recent paper reported an increased neoantigen-specific CD8^+^ T-cell response in marrow-infiltrating lymphocytes from pediatric patients with ALL, including a neoantigen from the good prognosis biomarker ETV6-RUNX1, evidencing a responsiveness of immune system in ALL patients carrying this fusion gene [12]. Neoantigens are self-antigens produced in tumor cells that result from acquired somatic mutations during the cells malignant transformation, like chromosomal translocations, point mutations, and insertions and deletions (indels). Neoantigens can also derive from unique proteins or peptides produced by dysregulated RNA splicing, disordered post-translational modification and integrated viral open reading frames [13]. These neoantigens can be presented by human leukocyte antigen (HLA) molecules of malignant cells for T-cell recognition to initiate an anticancer immune response in patients [25,26,27,28]. Identification of candidate neoantigens with high-throughput genomics and bioinformatics technologies is generally based on the standard approach in which affinity of the putative peptides encoded by the identified mutations, for a given HLA I allele, can predict T-cell response [18,29,30,31]. Discovering unique tumor-specific neoantigens in ALL offers an enormous opportunity for more effective personalized treatment implementation. Considering that mutated gene products can act as tumor neoantigens, by exome sequencing of normal-tumor paired samples from pediatric patients we explored the landscape of coding somatic mutations in ALL to identify potential neoantigens associated with OS.

## 2. Results

### 2.1. Clinical Characteristics of Studied Population

Sixty-four children with de novo ALL were included. Saliva samples and bone marrow (BM) were collected at diagnosis; 35 of them were matched normal (saliva)-tumor (BM) tissues. Overall, 58% were male and 42% female, median age of the population was 104.2 months at diagnosis (range: 6.48–202.36 months) and 88% of patients were diagnosed with pre-B immunophenotype. Fourteen (22%) patients presented relapse and 12 (19%) died. The median of the follow-up of the patients was 72 months (range: 60–84 months) after diagnosis confirmation (Table 1).

### 2.2. Distribution of HLA Class I Alleles

**Figure 1:**
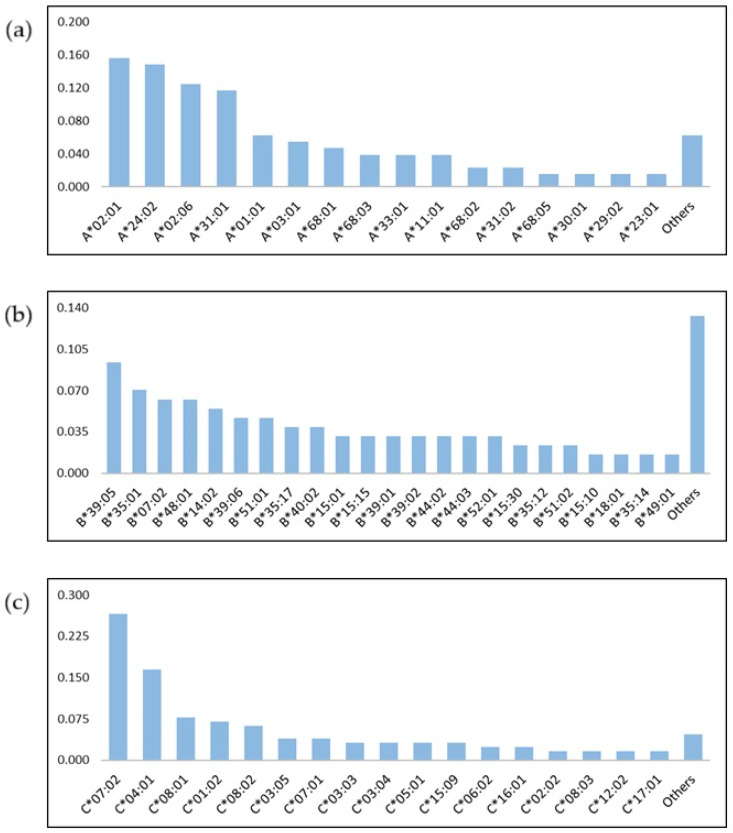
Frequency of HLA alleles in pediatric patients with acute lymphoblastic leukemia. HLA–A* (**a**), HLA–B* (**b**) and HLA–C* (**c**) alleles.

HLA genotype frequencies data were estimated for 64 ALL patients, of which 24 HLA–A*, 40 HLA–B* and 23 HLA–C* different alleles were identified. The most common HLA-A alleles in the ALL cohort were HLA-A*02:01 (15.6%) and HLA-A*24:02 (14.8%) present in 10 and 9 of the 64. Among HLA-B and -C alleles, HLA-B*39:05 (9.4%), HLA-B*07:02 (7%), HLA-C*07:01 (26.6%) and HLA-C*04:01 (16.4%) were the most frequent (Figure 1a–c). We evaluated the correlation among these six most frequent alleles with overall survival (OS) and event free survival (EFS), where event is defined as relapse or death, but no statistically significant associations were found (Table S1).

### 2.3. High Correlation of Missense Mutation and Neoantigen Load

The identification of potential neoantigens was carried out by including 35 paired normal-tumor samples. A total of 33,315 somatically acquired missense mutations were identified. A total of 9880 neoantigens were predicted by considering those peptides that could bind to at least one of the patients’ HLA-A, -B, or -C alleles with an affinity < 500 nM or %Rank < 2%. Even though neoantigens must ultimately be validated for their presentation and recognition by T lymphocytes, we will use the term “neoantigens” to describe “potential neoantigens” throughout the text. A single mutation can generate multiple neoantigens by binding to diverse HLA alleles or in distinct registers; nevertheless, predicted neoantigens were excluded if they displayed the same affinity binding to HLA as their wild-type counterparts. The number of neoantigens ranged from 0 to 4385, with a median of 47 *per* patient. Among these, the most common were EELRLDHPA, EHLDLDFSI, ERGLRWLVT, LAICLACSL, LRLDHPAMA, LYQGQLNKL, RALEIQPGL, SDDNSASLL, THKSDIYSF, TVVAVDRYM, YSKEHLAMM and YYRAHIAQL, which had frequencies ranging from 6% to 9%. ALL patients had low percentage of shared neoantigens (6–9%). We further assessed expression of *SYNGAP1*, *CUL1*, *COX11*, *PORCN*, *CPA2*, *PEX5L*, *TRAF3IP1*, *IRAK4*, *RRH*, *CLK4*, *CLK1*, *CLTCL1* genes in which the most frequent neoantigens were identified. We compared ALL patients and healthy subjects, from data retrieved by the TARGET initiative, RNA-seq level 3 data from cBioPortal [32] and GTEx project. Except for *PORCN*, all genes were differentially expressed among cases and controls (Figure S1). We identified sixteen HLA-corresponding alleles for each shared neoantigen and most of them belonged to HLA-B (9 alleles, 56%) and HLA-C (6 alleles, 37%); HLA-B*39:06 and HLA-C*07:02 alleles were found to bind to four neoantigens each. Only one allele corresponded to HLA-A (*24:02) group (Table S2).

**Figure 2:**
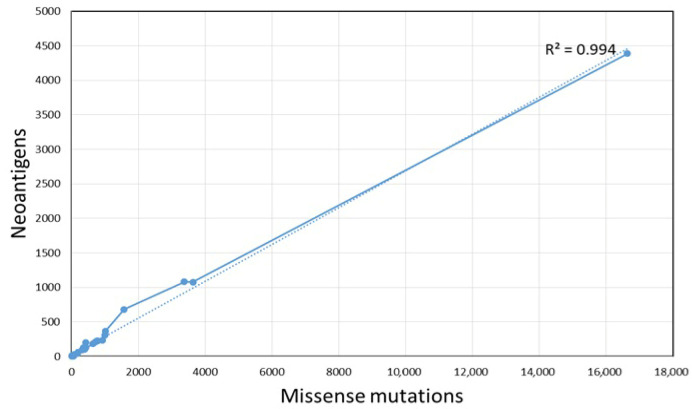
Correlation of mutation burden and the number of potential neoantigens.

Notably, we found that the number of neoantigens was positively correlated with the number of missense mutations (R = 0.994, *p* < 0.001) in ALL pediatric patients (Figure 2).

### 2.4. Association Among Clinical Features, Mutations and Neoantigens Numbers

When analyzing by gender, we found that male presented more mutations and neoantigens than female (X = 1301 vs. 687 and X= 360 vs. 217, respectively) but it did not reach statistical significance (*p* = 0.88 and 0.78; Table 2), whereas white blood cell (WBC), % of blasts in BM and risk stratification analyses showed no statistical significant differences, except for immunophenotype. Patients with T-cell ALL showed more missense mutations (X = 1991.7 vs. 872, *p* = 0.019) and neoantigens (X = 661.7 vs. 247.2, *p* = 0.025) (Table 2, Figure S2) but significance did not remain after permutation analysis.

### 2.5. Number of Mutations, Neoantigens and Neoantigen Frequency Survival Analysis

Patients were stratified in two groups to determine the association between the number of mutations (median = 174) and neoantigens (median = 47) compared to EFS and OS. We found no significant differences between them. As it has been previously described that low number of neoantigenic mutations could be used as an independent prognostic factor for clinical outcome in other cancers [33,34,35], we assessed the ratio of neoantigens per missense mutations and evaluated its association with EFS or OS. No statistically significant differences were found (Figure S3).

### 2.6. High Correlation Between Number of Mutations in DNA Mismatch Repair Genes and Neoantigens Load

**Figure 3:**
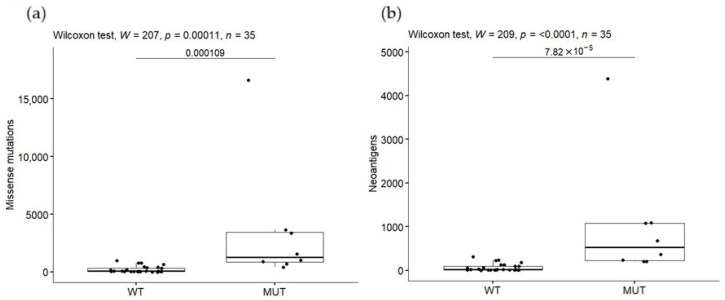
Mutational burden (**a**) and tumor neoantigen load (**b**) in patients carrying missense mutations in DNA mismatch repair genes.

As was previously documented, MMR-deficient cancers present a hypermutable state, which could generate a greater number of neoantigens that might be recognized by the immune system [26,36,37]. To test whether mutated DNA MMR genes could be implicated in an increase in number of mutations and consequently of neoantigens in ALL, we explored the mutations in *MSH2*, *MSH6*, *MLH1* and *PMS2*. A total of 29 mutations were detected in 8 (23%) of the 35 patients; 2 (9%) of them had mutations in *MSH2*, 2 (6%) in *MSH6*, 8 (23%) in *MLH1* and only 1 case (3%) in *PMS2* (Table S3). Patients were stratified in two groups: (a) presented at least one mutation in one of these genes (MUT) and (b) presented no mutations (WT). The MUT group displayed higher mutational burden (Figure 3a) and neoantigen load (Figure 3b) than the WT group (*p* < 0.001) and statistical significance remains after the permutation test (*p* = 0.002 and *p* = 0.0008 for mutations and neoantigens, respectively). Notably, the patient carrying the highest mutation burden (n = 16,633) and neoantigen load (n = 4385) was the one who harbors mutations in most of the DNA mismatch repaired evaluated genes (12/29 in three genes) (Figure 3). No associations were found among number of mutations in these genes and EFS and OS (Figure S4).

## 3. Discussion

The development of immunotherapy in recent years for ALL treatment has been focused on tumor-associated antigens which are overexpressed in leukemic cells but also expressed by normal cells in lower levels or in a specific stage or condition [38,39,40]. For instance, CD19 which is highly expressed during B-lineage neoplastic transformation, is a target for blinatumomab, a bispecific antibody, and for Tisagenlecleucel, a chimeric antigen receptor (CAR)-T cells therapy [41,42,43]. Nevertheless, this protein is also expressed during the differentiation of the hematopoietic stem cells, and its expression continues through B-cells lineage differentiation [44]. Additionally, CAR-T cells therapy still faces some challenges, such as reduced effectiveness in relapsed or chemoresistant patients, and high toxic side effects (cytokine release syndrome, B-cell aplasia, etc.). In fact, neurotoxicity for treatments based on a combination of bispecific antibodies and CAR-T has been observed [43,45,46]. Thus, discovering unique tumor-specific proteins in leukemic cells offers an enormous opportunity for more effective treatments. Even though it has been described that hematologic malignancies have fewer neoantigens than solid tumors [18,23,24], the immune response in allogenic hematopoietic stem cell transplantation and in immunotherapy for relapsed or refractory ALL [22] sets the basis to hypothesize that leukemic cells could express new immunogenic specific peptides derived from acquired coding mutations. Accordingly, we determined the missense mutational burden and neoantigen load in pediatric cases with ALL. Based on the germline exome data, we determined the frequency of HLA-A, -B and -C alleles in our ALL pediatric patients; further, we predicted the neoantigens with high binding affinity (affinity < 500 nM or %Rank < 2%) to patients’ HLA alleles.

In HLA-I alleles distribution, A*02:01, A*24:02, B*35:01, B*39:05, C*07:02 and C*04:01 were among the most frequent alleles as has been reported in Mexican ethnic groups like Tarasco, Tarahumara and Mestizo [47,48,49,50,51,52], as well as ALL cases [53,54]. Identifying the most common HLA alleles could be relevant for developing vaccines against human malignancies. For instance, the most frequent HLA-A alleles in our study, HLA-A*02:01 and -A*24:02, have been previously shown to be targeted for immunogenic approaches in cancer [55,56]. Both alleles have been associated with a variety of clinical outcomes in several diseases, but no association was found among them and clinical and demographic characteristics was identified in the present analysis [55,57,58,59,60].

A correlation between high missense mutational burden and predicted neoantigens load was found. Interestingly, our data differs from those published previously. First of all, a study of 100,000 human cancer genomes reported that pediatric malignancies, including pediatric ALL, had low abundance of somatic mutations (tumor mutational burden) [61]. Secondly, the coding mutations of this hematologic malignancy have been poorly explored as sources of neoantigens. To our knowledge, the only existing study was carried out by Zamora, whom by screening tumor-specific somatic missense mutations and gene fusions for their potential to generate neoantigens (strong putative neoantigens if IC_50_ < 150 nM, intermediate to weak < IC_50_ 150–500 nM and as putative nonbinders > 500 nM) found few predicted neoantigens per patient (2–23) [12]. Discrepancies among our study and Zamora’s findings could be associated with the data source (RNASeq) and neoantigen filtering criteria. Our study not only includes neoepitopes with affinity (IC50 < 500 nM) values as Zamora’s, but also, we consider the percent rank (<2%) score of binding affinity and the difference in binding affinity of the mutant peptide to its wild-type counterpart (differential agretopic index: DAI). Rank score is less biased than binding affinity when comparing binding between multiple HLA alleles and candidate neoantigens; rank < 2% have higher probability to be presented by the MHC-I molecule than neopeptides with percent rank score > 2 [62]. High DAI score is related to the ability of neoantigens to protect against the tumor [31,63,64]. In addition, we selected nonamer peptides, while Zamora’s included wider peptides (14aa); also, most of the included cases by Zamora were ETV6-RUNX1 positive (7/9). Due to the presence of common coding mutations and/or fusions genes (ETV6-RUNX1, E2A-PBX1, BCR-ABL, etc.) within subgroups of patients, it has been reported that hematologic malignancies have more shared neoantigens than most solid tumors [24,65]. Contrary to what was expected, shared neoantigens in our cohort were lower than private or personal neoantigens. Even when data of fusion genes for our patients was not available, the frequency of the highest shared predicted neoantigen was around the same reported for children ETV6-RUNX1 positive (7.4%) with ALL from Mexico [66]. Even predicted neoantigens were heterogeneous across different patients; shared neoantigens such as EELRLDHPA, EHLDLDFSI, ERGLRWLVT, LAICLACSL, LRLDHPAMA, LYQGQLNKL, RALEIQPGL, SDDNSASLL, THKSDIYSF, TVVAVDRYM, YSKEHLAMM and YYRAHIAQ could be promising candidates for the development of immunotherapy regimens based on neoantigen vaccines. The aim of these vaccines is training the immune system against tumor cells in patients which are positive to tumor-specific neoantigens [67,68]. Neoantigen vaccines can be developed using peptides, mRNA, DNA and dendritic cells. Although these neoantigen vaccines could act as individualized drugs, combinations with chemotherapy, radiotherapy, immune checkpoint inhibitors, etc., might improve the efficacy of anticancer treatments based on tumor-specific neoantigens [67]. In addition to ALL and accounting that the identified neoantigens in this study were located in genes previously associated with other malignancies (breast cancer, melanoma, non-small cell lung cancer, etc.), we cannot discard their potential clinical application in the treatment of those tumors [69,70,71,72,73,74,75,76].

After in silico testing of whether the neoantigens can be presented by the MHC using computational prediction tools, additional steps are needed to know if the predicted neoantigens can be recognized by T-cells and induce an immune response: (a) assessing peptide transport from the cytoplasm to the endoplasmic reticulum mediated by transporters associated with antigen processing is a crucial process in the intracellular presentation of cytotoxic T-cell epitopes [77]; (b) determining the proteins’ or peptides’ expression abundance, either by evaluating the source of the proteins, the gene expression, or peptides’ expression by mass spectrometry analyses [78,79,80,81]; and (c) functional analysis to test whether the neoantigen can be recognized by a T-cell receptor and trigger T-cell activation. These tests could be made through assays as enzyme-linked immunosorbent spot (ELISPOT), which measure cytokine release, supported lipid bilayer (SLB)-based T-cell activation that allows monitoring interactions between the neoantigen-HLA and the T-cells and DNA barcode-based neoantigen–HLA tetramers and flow cytometry to identify the neoantigen–HLA–T-cells bounds [82,83,84,85]. Besides this, the expression levels of those genes having potential neoantigens were explored in ALL samples and normal tissue. RNA-seq data from cases and controls were retrieved from the TARGET initiative, RNA-seq phase 2 [32] and the GTEx project, respectively. Comparative analysis between cases and controls showed that except for *PORCN*, all genes were differentially expressed among ALL patients and controls, which increases the possibility of antigen expression abundance.

Another interesting finding was the relation among the higher mutational burden and neoantigen load with the presence of mutations in the DNA MMR genes *MSH2*, *MSH6*, *MLH1* and *PMS2.* Patients negative for mutations in these genes have lower mutational burden and neoantigen load than the positive ones (X = 3532.25 vs. 1028 and 297.47 vs. 89.06, respectively; *p* = 0.0001 and 0.00007). These discoveries are similar not only to those previously documented in solid tumors such as colorectal cancer and lung adenocarcinoma but also to pediatric cancers [26]. Overall, *MLH1* (41.5%) was the most commonly mutated DNA MMR gene, followed by *MSH6* (34.5%) and *MSH2* (21%). DNA MMR genes, particularly *MLH1* and *MSH2,* have an important role in maintaining replication fidelity; thus, mutations in these genes lead to genome instability by not correcting base mismatches generated during DNA replication and cancer development [86]. This is particularly relevant since it has been shown that mutations in DNA MMR genes are sensitive to immune checkpoint blockade, even in healthy cells [36].

The association analysis among neoantigen frequency per mutation and clinical (WBC, immunophenotype, EFS and OS) variables showed no statistical differences among groups, which could be explained by the sample size. Since it has been reported that mutational burden and neoantigen load is correlated with prognosis in diverse cancer types (i.e., melanomas, non-small cell lung cancers and colorectal cancer, etc.) [87,88,89,90,91], predicted neoantigens identified in this study and preferentially those found in higher frequencies must be validated for their presentation and recognition by T lymphocytes to be named as neoantigens. To be proposed as neoantigens and potential therapeutic targets and considering their relevance in immunotherapy for ALL, predicted neoantigens must be tested in vitro.

## 4. Materials and Methods

### 4.1. Patients, Samples and Data

Samples of tumor (BM) and normal (saliva) tissues of ALL pediatric patients which were collected at diagnosis and before clinical treatment were included. To identify somatic mutations, which were defined as variants present in tumor tissue but not in normal tissue of the patient, we obtain germline DNA through saliva samples. All patients were under 18 years old and recruited from 2014 to 2016, with a follow-up of >36 months after diagnosis confirmation. Cases were residents of the metropolitan area of Mexico City and recruited from public hospitals and health institutions from Mexico City by the Mexican Interinstitutional Group for the Identification of the Causes of Childhood Leukemia (MIGICCL). ALL diagnosis was confirmed by pediatric hematologists or oncologists according to morphology and immunophenotype of leukemic cells. Clinical data such as gender, age at diagnosis, WBC count, immunophenotype, risk of relapse, relapse and death were registered from the patients’ medical records. Risk stratification criteria from The National Cancer Institute (NCI) was employed. Standard risk: from 1 to 9.99 years of age or WBC count < 50 × 10^9^/L, and high risk: ≤1 or ≥10 years of age and/or WBC ≥ 50 × 10^9^/L. Relapse was defined as disease recurrence in patients who had previously achieved morphologic remission, characterized by <5% blasts in bone marrow and clearance of extramedullary disease. Cases with Down syndrome were excluded from the analysis. This study was approved by the National Ethics and Research Committees of the Instituto Mexicano del Seguro Social (IMSS) (R-2015-785-121). Written informed consent was obtained from the children’s parents and children assented when possible.

### 4.2. DNA Isolation and Exome Sequencing

Germline DNA from saliva was isolated using prepIT.L2P ORAGENE Purification Kit (DNA Genotek Inc., Kanata, ON, Canada). Tumor DNA was obtained from mononuclear cells, which were isolated from BM mononuclear by density gradient centrifugation and using the Gentra Puregene Blood Kit (Gentra Systems Inc., Minneapolis, MN, USA) according to the manufacturer’s instructions. DNA purity and concentration were determined by Nanodrop spectrophotometer ND1000 (Thermo Fisher Scientific, Waltham, MA, USA).

Paired-end whole-exome sequencing (WES) was performed with Illumina NEXSeq, kit NexteraXT as previously described [92] and a depth of 100X. The sequenced raw data were aligned against hg38 human genome using the Burrows–Wheeler Aligner (BWA) [93,94]. In addition, removal of duplicate reads, SNPs and InDels realignment, and Base Quality Score Recalibration were performed using the Genome Analysis Toolkit (GATK), as previously described [95,96]. Mutations were annotated with Variant Effect Predictor (VEP, https://www.ensembl.org/info/docs/tools/vep/index.html, accessed on 1 February 2022) and MuTect (https://gatk.broadinstitute.org, accessed on 1 January 2022) was used to identify variants on processed data.

### 4.3. Class I HLA Alleles Prediction and Peptides–HLA Binding Predictions

Optitype (https://github.com/FRED-2/OptiType, accessed on 5 April 2021) with default settings was used for prediction of class I HLA alleles with 97% accuracy [97].

Tumor-specific missense mutations were identified to generate an amino acid sequence of potential neoantigens. Peptides covering nine tiling nonamers overlapping each missense mutation were extracted through a program designed with the SeqIO module of Biopython [98]. We tested peptide binding affinity with each of the patient’s HLA class I alleles by using NetMHCcons v1.1 program [99]. Predictions were made in May 2023, using the latest version of the algorithm at the time. Peptides were defined as candidate epitopes based on the affinity definition as half maximal inhibitory concentration (IC_50_) of <500 nM or % Rank < 2% of prediction score to a set of 200,000 random natural 9mer peptides for any HLA. Predicted binders, whether strong or intermediate, were considered as potential neoantigens.

### 4.4. Gene Expression Analysis of the Most Frequent Neoantigens

To know whether *SYNGAP1*, *CUL1*, *COX11*, *PORCN*, *CPA2*, *PEX5L*, *TRAF3IP1*, *IRAK4*, *RRH*, *CLK4*, *CLK1* and *CLTCL1* genes are expressed in ALL, RNA-seq data from ALL cases and controls were retrieved from the TARGET (Therapeutically Applicable Research to Generate Effective Treatments) initiative, RNA-seq level 3 [32] and The GTEx (Genotype-Tissue Expression) project (https://gtexportal.org/home/, accessed on 19 August 2025), respectively. The TARGET cohort consists of 463 ALL cases (phs000464) and GTEx of 407 non-cancerous subjects. Comparative analysis between cases and controls was performed using the TNMplot platform [100].

### 4.5. Mutations in DNA Mismatch Repair Genes

Based on the knowledge that genes involved in DNA MMR are associated with the number of neoantigens and favorable prognosis in diverse types of cancer [17,101], we explored the landscape of *MSH2*, *MSH6*, *MLH1* and *PMS2* genes through exome sequencing data.

### 4.6. Statistical Analysis

Statistical analyses were performed in R-Studio (R version 4.3.2) [102]. The Wilcoxon–Mann–Whitney tests were calculated to identify the statistical significance of differences between comparative groups regarding distribution of clinical and molecular characteristics. A *p*-value < 0.05 was considered statistically significant. Permutation test was performed by using lmPerm package (version 2.1.4). Default settings were used, in which iteration number reached 5000, and/or the standard error of the estimate of the *p*-value goes below the threshold (default = 10% of *p*-value). Linear regression analysis was performed to evaluate correlation between number of mutations and number of potential neoantigens. The hazard ratio was determined using a Cox proportional model to describe the effect of neoantigen burden in patients’ outcome. Correlations between nonsynonymous mutation burden and neoantigen burden, neoantigen burden and overall response were calculated using Spearman correlation formula.

## 5. Conclusions

In this study, we observed a high correlation between mutational burden and antigens load in ALL pediatric cases. Our data favors immunotherapy based on the discovery of neoantigens as an opportunity for developing new ALL treatment protocols in pediatric patients, particularly for treating ALL relapse, which can substantially reduce sequelae derived from conventional chemotherapy.

## Figures and Tables

**Table 1:** Clinical characteristics of pediatric patients with acute lymphoblastic leukemia.

Clinical Characteristics	Acute Lymphoblastic Leukemia Patients *n* = 64	Normal-Tumor Paired Patients *n* = 35
Gender		
Male	37 (58%)	19 (54%)
Female	27 (42%)	16 (46%)
Age (months)	104.2 (±61.4)	104.5 (±62.2)
Immunophenotype		
Pre-B	56 (88%)	32 (91%)
T-cell	8 (12%)	3 (9%)
Leukocytes in PB (≥50,000 mm^3^)	19 (30%)	10 (29%)
NCI risk classification		
High risk	25 (39%)	14 (40%)
Standard risk	38 (64%)	21 (60%)
Relapsed	14 (22%)	4 (11%)
Death	12 (19%)	5 (14%)

**Table 2:** Correlation mutations and neoantigens in clinical features groups.

Clinical Features	Missense Mutations (Mean)	*p*-Value	Neoantigens (Mean)	*p*-Value
Sex		0.882		0.778
Female	1301		360.6	
Male	687.5		217.2	
Age (years at diagnosis)		0.741		1
<10 y	1256.4		348.9	
>10 y	695.5		220.2	
WBC/ µL at diagnosis		0.235		0.235
<50,000	1039.12		289.84	
>50,000	790		264.9	
Blast % in BM at diagnosis		0.517		0.717
<50%	222		69.5	
>50%	1064.2		310.2	
Immunophenotype		0.019 *		0.025 *
B-cell precursor	872		247.2	
T-cell	1991.7		661.7	
NCI risk classification		0.381		0.590
Standard	664.9		211.8	
High	1422.6		389.1	

* Statistically significant. WBC: white blood cell, BM: bone marrow, NCI: National Cancer Institute.

## Data Availability

The data are not publicly available because they are protected by the Instituto Nacional de Medicina Genómica; however, data are available on reasonable request to the corresponding author.

## Supplementary Materials

The following supporting information can be downloaded at: https://www.mdpi.com/article/10.3390/ph18091405/s1, Table S1: Association of most frequent HLA-I alleles with relapse and death; Table S2: Neoantigens with higher frequency; Table S3: Neoantigens and mutations in ALL patients with mutated and non-mutated MMR genes; Figure S1: Expression boxplots of genes in which potential neoantigens are located. (A) *SYNGAP1*, (B) *CUL1*, (C) *COX11*, (D) *PORCN*, (E) *CPA2*, (F) *PEX5L*, (G) *TRAF3IP1*, (H) *IRAK4*, (I) *RRH*, (J) *CLK4*, (K) *CLK1*, (L) *CLTCL1*; Figure S2: Mutations and neoantigens in clinical features groups; Figure S3: Numbers of mutations, neoantigens and frequency of neoantigens are not correlated with prognosis in ALL patients. A, Kaplan–Meier event-free (left) and overall (right) survival curves in 35 ALL patients stratified according to the number of mutations. B, Kaplan–Meier event-free (left) and overall (right) survival curves in 35 ALL patients stratified according to the number of neoantigens. C, Kaplan–Meier progression-free (left) and overall (right) survival curves for ALL patients stratified according to the frequency of neoantigens per missense mutation; Figure S4: Numbers of mutations in MMR genes are not correlated with prognosis in ALL patients. Kaplan–Meier event-free (left) and overall (right) survival curves in 35 ALL patients stratified according to the number of mutations in MMR genes. MUT: at least one mutation in one, WT: no mutation.

## Abbreviations

The following abbreviations are used in this manuscript:

ALL	Acute Lymphoblastic Leukemia
TMB	Tumor Mutational Burden
OS	Overall Survival
MMR	Mismatch Repair
HLA	Human Leukocyte Antigen
EFS	Event Free Survival
WBC	White Blood Cells
BM	Bone Marrow
WT	Wild-Type, Non-Mutated
MUT	Mutated
CAR	Chimeric Antigen Receptor
MIGICCL	Mexican Interinstitutional Group for the Identification of the Causes of Childhood Leukemia
NCI	The National Cancer Institute
