# Supplementary material for: Somatic Mutations in DNA Mismatch Repair Genes, Mutation Rate and Neoantigen Load in Acute Lymphoblastic Leukemia
Source: Pharmaceuticals (Basel). 2025 Sep 18;18(9):1405. doi: 10.3390/ph18091405 (PMC12472650; doi:10.3390/ph18091405)
Supplement: Supplementary file 1 [file pharmaceuticals-18-01405-s001.zip › pharmaceuticals-3808694-supplementary.pdf]

Supplementary Information

Table S1. Association of most frequent HLA-I alleles with relapse and death.

Alelle	Relapse			Death		
	OR(95%IC)	X^2	p-value	OR(95%IC)	X^2	p-value
HLA-A*24	0.79(0.17-3.71)	0.09	0.77	0.95(0.20-4.55)	0.00	0.95
HLA-A*02	0.43(0.10-0.18)	1.36	0.24	0.54(0.13-2.34)	0.69	0.41
HLA-A*31	0.79(0.17-3.71)	0.09	0.76	3.14(0.70-14-13)	2.33	0.13
HLA-B*39	1.5(0.36-6.18)	0.32	0.57	1.09(0.25-4.75)	0.01	0.91
HLA-C*07	0.76(0.18-3.21)	0.13	0.71	0.27(0.05-1.48)	2.46	0.12
HLA-C*04	0.34(0.06-1.90)	1.58	0.21	0.56(0.12-2.60)	0.54	0.46

Table S2. Neoantigens with higher frequency.

ID	HLA haplotype	Variant	Mutated gene	Predicted high affinity neoepitope	Corresponding HLA	Predicted affinity (IC_{50}/%Rank)
LLA-10	A*02:01 A*68:03<br>B*18:01 B*39:05<br>C*07:01 C*07:02	SYNGAP1_S44A	*SYNGAP1*	EE**L**RLDHPA	HLA-B18:01	579.42/1.0
LLA-31	A*24:02 A*68:01<br>B*39:06 B*40:02<br>C*03:05 C*03:05				HLA-B40:02	324.79/1.50
LLA-16	A*02:06 A*68:01<br>B*39:01 B*39:05<br>C*07:02 C*07:02	CUL1_P530H	*CUL1*	E**H**LDLDFSI	HLA-B39:01<br>HLA-B39:05	97.20/0.80<br>198.92/0.30
LLA-28	A*24:02 A*31:01<br>B*14:02 B*39:06<br>C*07:02 C*08:02				HLA-B39:06	2990.60/1.50
LLA-20	A*02:06 A*33:01<br>B*14:02 B*35:17<br>C*04:01 C*08:02	COX11_G54V	*COX11*	ERGLRWL**V**T	HLA-B14:02	2751.98/1.50
LLA-28	A*24:02 A*31:01<br>B*14:02 B*39:06<br>C*07:02 C*08:02				HLA-B14:02	2751.98/1.50
LLA-21	A*31:02 A*31:02<br>B*15:30 B*35:01<br>C*01:02 C*04:01	PORCN_R40S	*PORCN*	LAICLAC**S**L	HLA-B15:30<br>HLA-B35:01<br>HLA-C01:02	654.01/1.50<br>379.96/3.00<br>3997.24/2.00
LLA-35	A*11:01 A*24:02<br>B*07:02 B*35:01<br>C*04:01 C*07:02				HLA-B35:01	379.96/3.00
LLA-07	A*01:01 A*24:02<br>B*27:05 B*39:06<br>C*01:02 C*07:02	CPA2_R415L	*CPA2*	LKAIMEHV**L**	HLA-B39:06	3711.18/2.00
LLA-10	A*02:01 A*68:03<br>B*18:01 B*39:05<br>C*07:01 C*07:02				HLA-B39:05	1125.75/1.50
LLA-30	A*68:01 A*68:03<br>B*39:05 B*48:01<br>C*07:02 C*08:01				HLA-B39:05<br>HLA-B48:01	1125.75/1.50<br>4406.07/2.00

LLA-28	A*24:02 A*31:01 B*14:02 B*39:06 C*07:02 C*08:02	SYNGAP1_R1189L	*SYNGAP1*	LR**L**DHPAMA	HLA-B39:06	3750.25/2.00
LLA-31	A*24:02 A*68:01 B*39:06 B*40:02 C*03:05 C*03:05				HLA-B39:06	3750.25/2.00
LLA-16	A*02:06 A*68:01 B*39:01 B*39:05 C*07:02 C*07:02	CLTCL1_D423Y	*CLTCL1*	L**Y**QGQLNKL	HLA-C07:02	837.07/2.00
LLA-18	A*02:01 A*24:02 B*35:17 B*48:01 C*04:01 C*08:03				HLA-A24:02	233.50/0.80
LLA-18	A*02:01 A*24:02 B*35:17 B*48:01 C*04:01 C*08:03	PEX5L_F539L	*PEX5L*	RALEIQPG**L**	HLA-B48:01	696.43/0.30
LLA-21	A*31:02 A*31:02 B*15:30 B*35:01 C*01:02 C*04:01				HLA-C01:02	2707.68/0.80
LLA-21	A*31:02 A*31:02 B*15:30 B*35:01 C*01:02 C*04:01	TRAF3IP1_R407L	*TRAF3IP1*	SDDNSASL**L**	HLA-C04:01	4625.90/0.50
LLA-28	A*24:02 A*31:01 B*14:02 B*39:06 C*07:02 C*08:02				HLA-C08:02	1672.99/1.00
LLA-03	A*02:01 A*03:01 B*07:02 B*52:01 C*03:03 C*07:02	IRAK4_P242H	*IRAK4*	T**H**KSDIYSF	HLA-C07:02	1097.07/2.00
LLA-30	A*68:01 A*68:03 B*39:05 B*48:01 C*07:02 C*08:01				HLA-C07:02	1097.07/2.00
LLA-06	A*01:01 A*11:01 B*27:02 B*52:01 C*02:02 C*12:02	RRH_L125M	*RRH*	TVVAVDRY**M**	HLA-C12:02	269.60/2.00
LLA-21	A*31:02 A*31:02 B*15:30 B*35:01 C*01:02 C*04:01				HLA-B35:01	386.17/3.00
LLA-21	A*31:02 A*31:02 B*15:30 B*35:01 C*01:02 C*04:01	CLK4_D381Y	*CLK4*	**Y**SKEHLAMM	HLA-B15:30 HLA-C01:02	672.11/1.50 3786.74/2.00
LLA-28	A*24:02 A*31:01 B*14:02 B*39:06 C*07:02 C*08:02		*CLK1*		HLA-C07:02	677.84/1.50
LLA-17	A*02:06 A*31:01 B*35:12 B*44:03 C*04:01 C*06:02	CLTCL1_D609Y	*CLTCL1*	Y**Y**RAHIAQL	HLA-C04:01 HLA-C06:02	2537.49/0.17 156.47/0.25
LLA-18	A*02:01 A*24:02 B*35:17 B*48:01 C*04:01 C*08:03				HLA-A24:02 HLA-C04:01	97.73/0.40 2537.49/0.17

Table S3. Neoantigens and mutations in ALL patients with mutated and non-mutated MMR genes.

ID	*MSH2*	*MSH6*	*MLH1*	*PMS2*	Missense mutations	Potential neoantigens
LLA-01					15	0
LLA-33					14	2
LLA-32					22	3
LLA-27					8	4
LLA-04					24	5
LLA-14					50	5
LLA-29					30	5
LLA-11					13	8
LLA-12					35	8
LLA-09					17	9
LLA-02					34	10
LLA-26					42	11
LLA-22					68	14
LLA-34					37	15
LLA-13					68	19
LLA-19					69	19
LLA-15					60	29
LLA-23					174	47
LLA-31					186	63
LLA-08					310	94
LLA-25					386	99
LLA-20					354	124
LLA-05					430	125
LLA-35					632	184
LLA-17			1		425	201
LLA-06			1		710	206
LLA-24					767	229
LLA-30					780	229
LLA-07			1		917	231
LLA-03					995	311
LLA-10	1		1	1	1004	362
LLA-16			4		1566	681
LLA-28		3	2		3629	1075
LLA-18	1		1		3374	1083
LLA-21	4	7	1		16633	4385

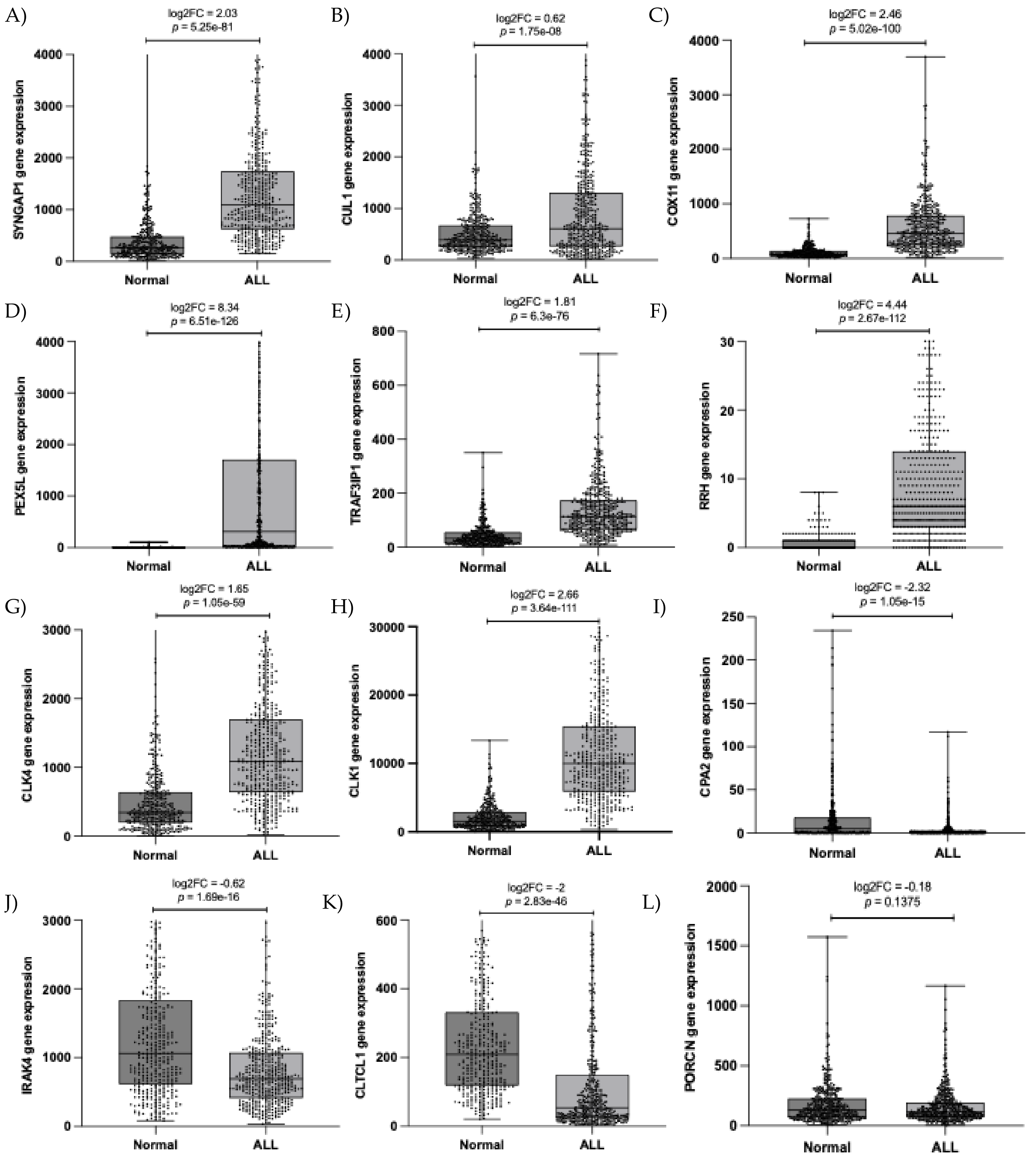

Figure S1. Expression boxplots of genes in which potential neoantigens are located. Upregulated A) *SYNGAP1*, B) *CUL1*, C) *COX11*, D) *PEX5L*, E) *TRAF3IP1*, F) *RRH*, G) *CLK4*, H) *CLK1*; Downregulated I) *CPA2*, J) *IRAK4*, K) *CLTCL1*, and with no difference L) *PORCN* in ALL patients.

A)

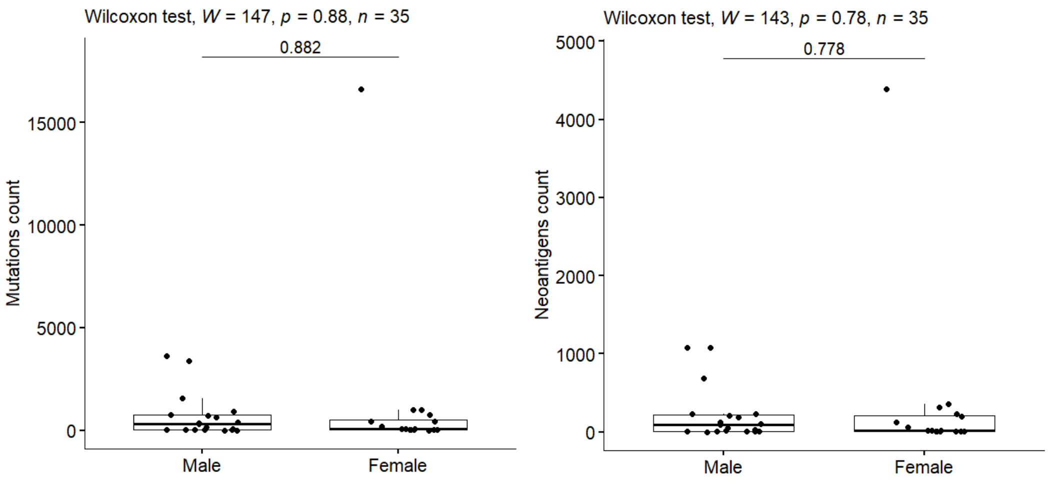

Wilcoxon test, W = 147, p = 0.88, n = 35
0.882
Mutations count
0
5000
10000
15000
Male
Female
Wilcoxon test, W = 143, p = 0.78, n = 35
0.778
Neoantigens count
0
1000
2000
3000
4000
5000
Male
Female

B)

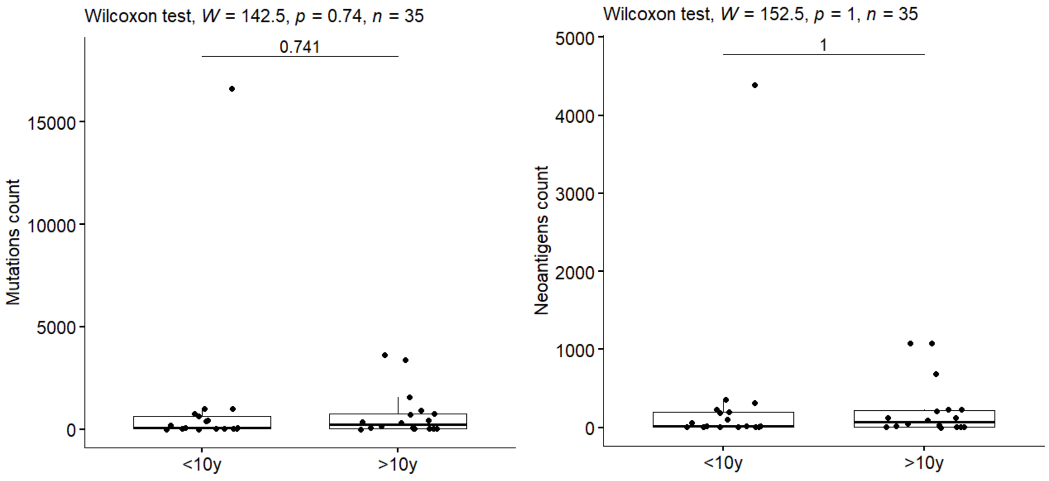

Wilcoxon test, W = 142.5, p = 0.74, n = 35
0.741
Mutations count
0
5000
10000
15000
<10y
>10y
Wilcoxon test, W = 152.5, p = 1, n = 35
1
Neoantigens count
0
1000
2000
3000
4000
5000
<10y
>10y

C)

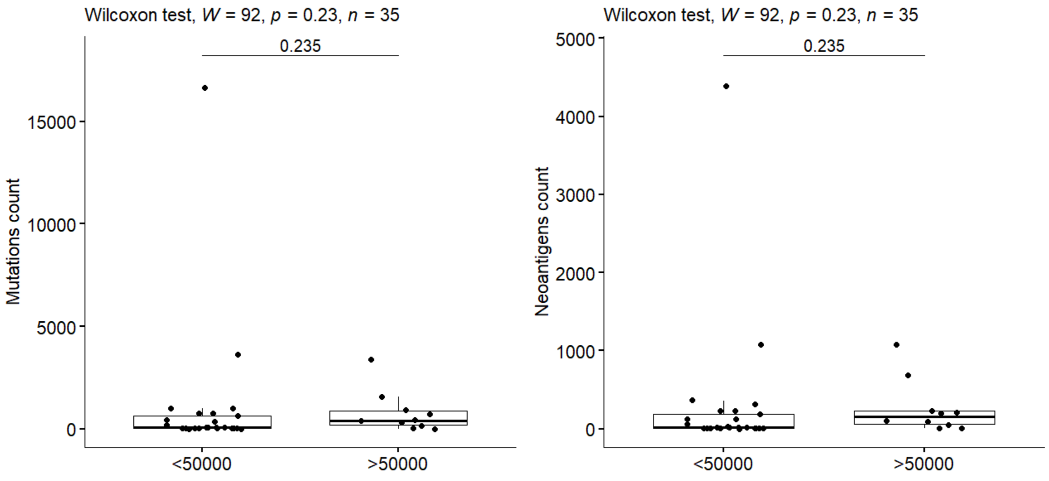

Wilcoxon test, W = 92, p = 0.23, n = 35
0.235
Mutations count
0
5000
10000
15000
<50000
>50000
Wilcoxon test, W = 92, p = 0.23, n = 35
0.235
Neoantigens count
0
1000
2000
3000
4000
5000
<50000
>50000

D)

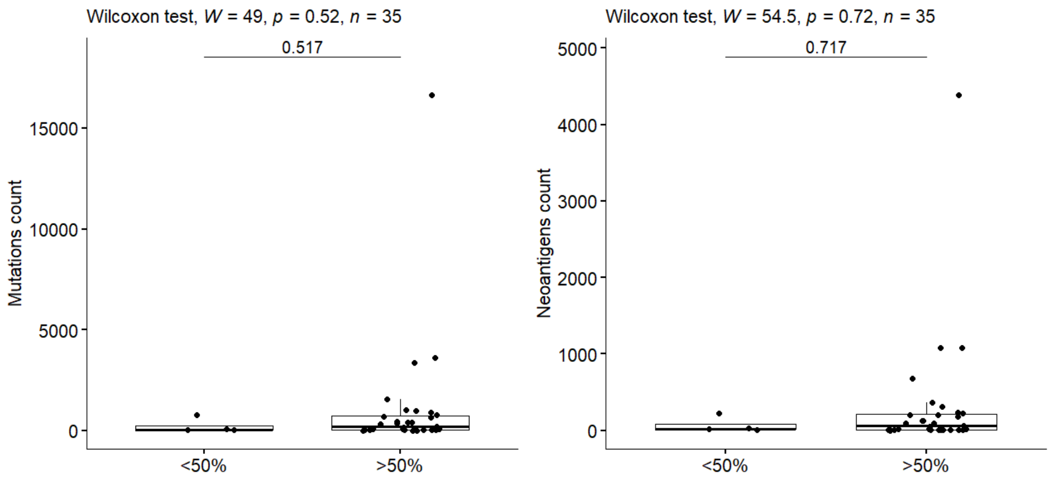

E)

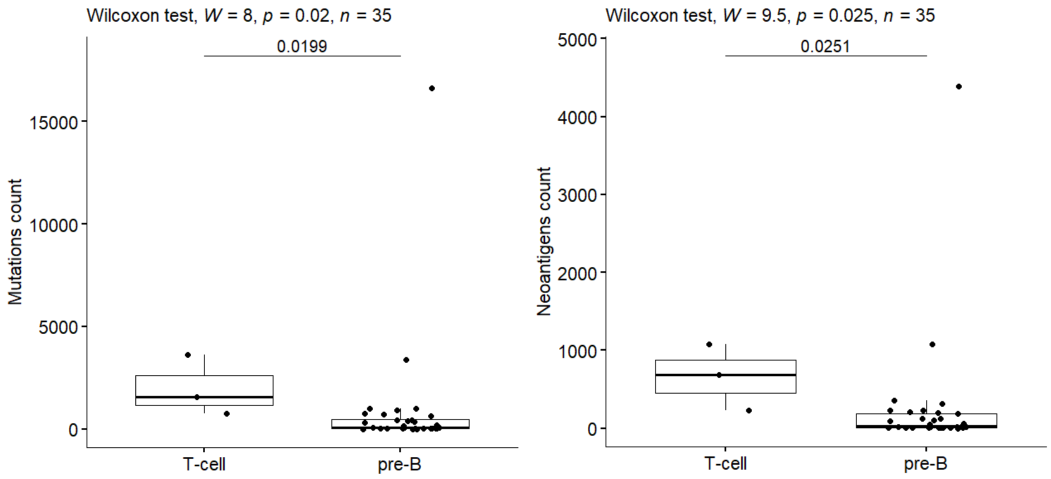

F)

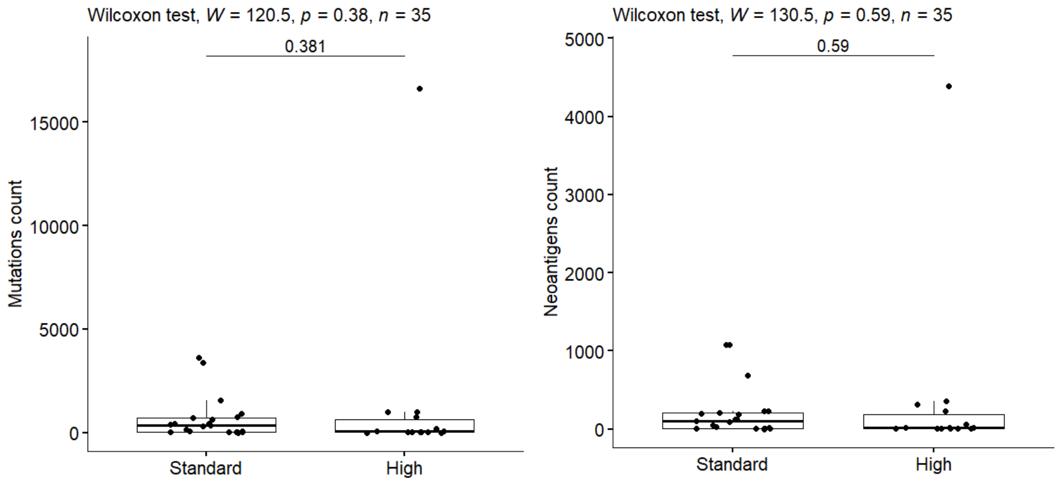

Figure S2. Mutations and neoantigens in clinical features groups. A) Gender, B) Age, C) Leukocytes in periferial blood, D) % blasts in bone marrow, E) Immunophenotype, F) NCI risk classification.

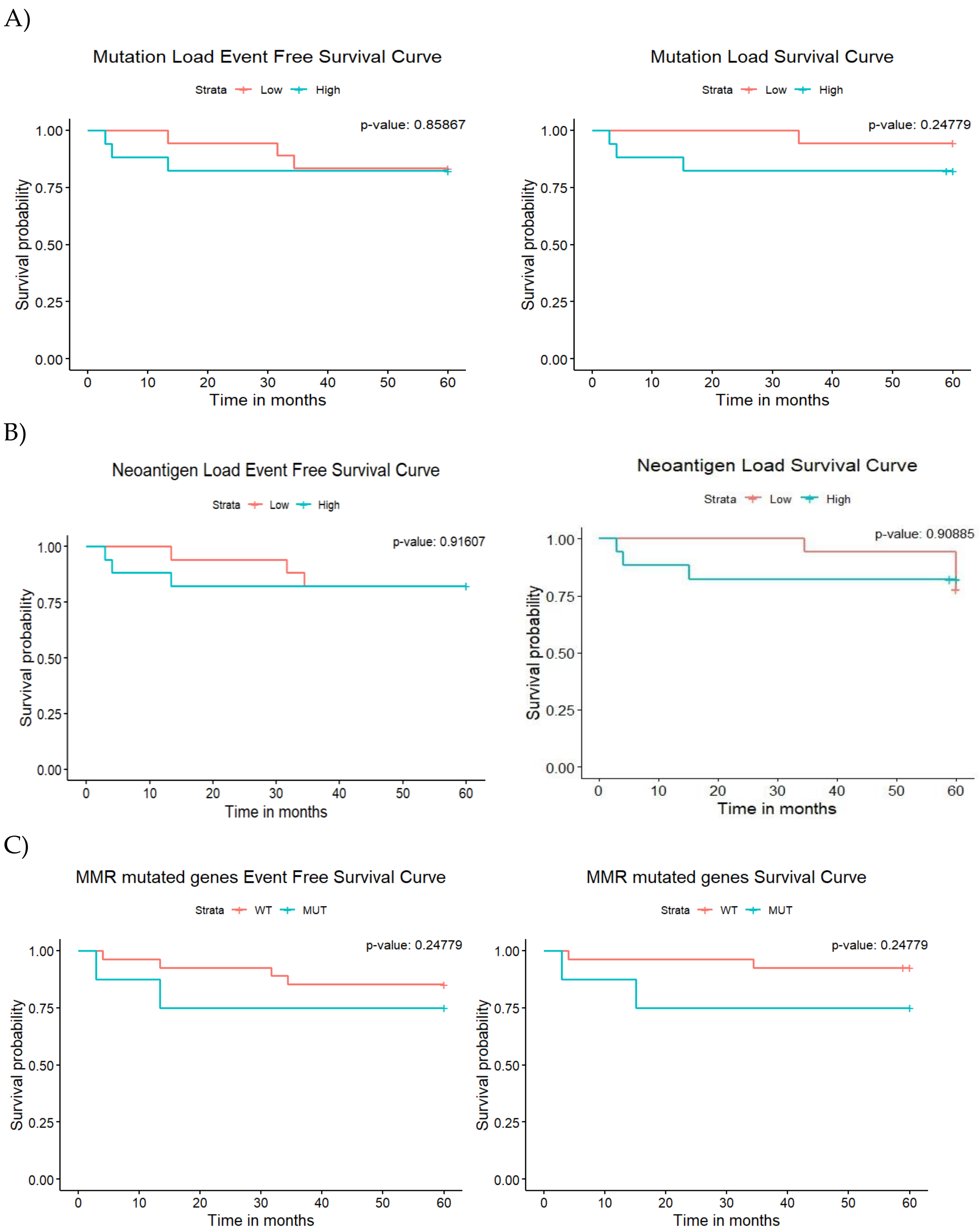

Figure S3. Numbers of mutations, neoantigens and frequency of neoantigens are not correlated with prognosis in ALL patients. A) Kaplan-Meier event-free (left) and overall (right) survival curves in 35 ALL patients stratified according to the number of mutations. B) Kaplan-Meier event-free (left) and overall (right) survival curves in 35 ALL patients stratified according to the number of neoantigens. C) Kaplan-Meier progression-free (left) and overall (right) survival curves for ALL patients stratified according to the frequency of neoantigens per missense mutation.

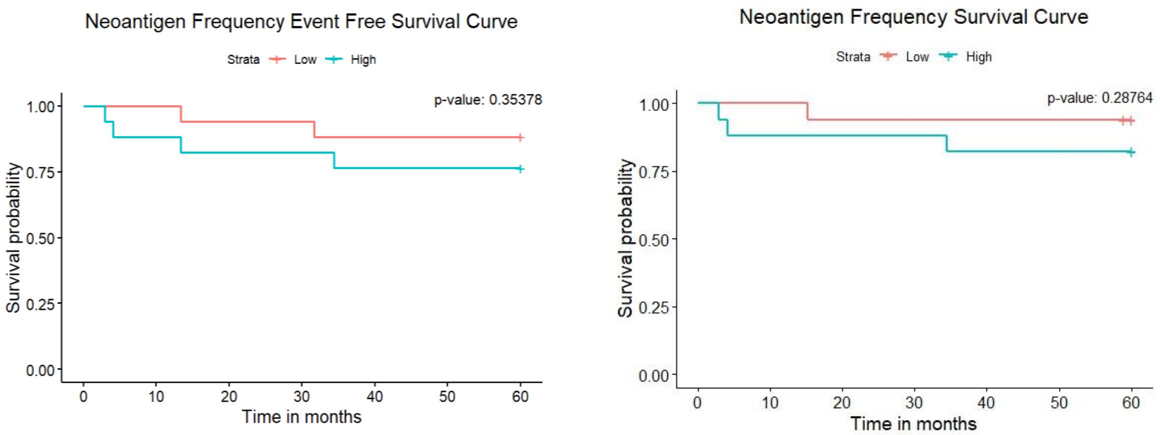

Figure S4. Numbers of mutations in MMR genes are not correlated with prognosis in ALL patients. Kaplan-Meier event-free (left) and overall (right) survival curves in 35 ALL patients stratified according to the number of mutations in MMR genes. ***MUT***: at least one mutation in one **WT**: no mutation.
